# Application of artificial intelligence in glioma researches: A bibliometric analysis

**DOI:** 10.3389/fonc.2022.978427

**Published:** 2022-08-11

**Authors:** Dewei Zhang, Weiyi Zhu, Jun Guo, Wei Chen, Xin Gu

**Affiliations:** The Department of Neurosurgery, Jing’an District Center Hospital of Shanghai, Fudan University, Shanghai, China

**Keywords:** glioma, bibliometric analysis, R studio, artificial intelligence, bibliometrix

## Abstract

**Background:**

There have been no researches assessing the research trends of the application of artificial intelligence in glioma researches with bibliometric methods.

**Purpose:**

The aim of the study is to assess the research trends of the application of artificial intelligence in glioma researches with bibliometric analysis.

**Methods:**

Documents were retrieved from web of science between 1996 and 2022. The bibliometrix package from Rstudio was applied for data analysis and plotting.

**Results:**

A total of 1081 documents were retrieved from web of science between 1996 and 2022. The annual growth rate was 30.47%. The top 5 most productive countries were the USA, China, Germany, France, and UK. The USA and China have the strongest international cooperative link. Machine learning, deep learning, radiomics, and radiogenomics have been the key words and trend topics. “Neuro-Oncology”, “Frontiers in Oncology”, and “Cancers” have been the top 3 most relevant journals. The top 3 most relevant institutions were University of Pennsylvania, Capital Medical University, and Fudan University.

**Conclusions:**

With the growth of publications concerning the application of artificial intelligence in glioma researches, bibliometric analysis help researchers to get access to the international academic collaborations and trend topics in the research field.

## Introduction

Gliomas are central nervous tumors that arise from glial or precursor cells, including astrocytoma (glioblastoma), oligodendroglioma, ependymoma, and some rare types (1). It is the second most common primary central nervous system (CNS) tumors (1). In 2016, the World Health Organization (WHO) classified gliomas into 4 grades and grade II-IV are considered to be infiltrative. It also emphasized that isocitrate dehydrogenase (IDH) and codeletion of 1p/19q were vital to clinical outcomes (2). While the newest WHO classification recommend that the diagnosis should rely on IDH-mutation and 1p/19q codeletion status because solid evidence has proved that the overall survival and therapeutic response vary drastically among different genetic subtypes (3–5). Genetic mutations play an important part in the prediction of the prognosis. However, genetic testing is costly and time consuming, an alternative, more economical and noninvasive approach for obtaining genetic information is needed, such as artificial intelligence (AI) methods.

ML (machine learning) is a discipline based on computer science and statistics which can recognize relationships from data iteratively (6). ML algorithms can be used to output a probability to predict prognosis and subtypes of a certain disease. With the rapid development of machine learning (ML) and deep learning (DL) technologies, features extracted from magnetic resonance imaging (MRI) can be included to construct models to predict the genetic mutations noninvasively (7–11). Prior knowledge of the genomic type will help patients with less aggressive tumors accept treatment at an appropriate time point. It also replaces the costly genetic sequencing. Thus, machine learning, radiomics, and deep learning technologies are important in glioma researches.

Notably, there is an increasing number of articles being published every year on glioma and AI. Therefore, it’s of vital importance for researchers to keep a constant and appreciable update of the latest literature. Bibliometric analysis is a kind of statistical method that depicts the knowledge structure and development trends of a specific research field with science mapping (12). Science mapping is complex and unwieldy because it is a multi-step process and frequently requires diverse software tools, which are not all freeware. Bibliometric analysis facilitates researchers to choose promising research directions. It also provides the active research institutions and groups which may promote potential international or national research cooperation. In the present study, we introduce a novel bibliometric analysis tool, “bibliometrix”. Bibliometrix is a package of Rstudio for the construction and visualization of the bibliometric network of the related literature (13). It is particularly suitable for science mapping at a time when the emphasis on empirical contributions is producing voluminous, fragmented, and controversial research streams. Bibliometric methods have been performed in different fields in medical researches (14). To the best of our knowledge, there has been no study for the overview of the application of AI in gliomas with bibliometric analysis. Herein, we performed an ML-based bibliometric method to provide an overview of the research trends concerning the application of AI in gliomas in the past 26 years.

## Materials and methods

### Search strategy

In May 2022, we conducted a literature search in web of science to collect relevant publications. Concerning the application of AI in glioma researches, we performed the following searching terms: (“glioma” OR “neuroglioma” OR “Intracranial glioma” OR “cerebral glioma” OR “gliomas” OR “neurogliomas” OR “intracranial gliomas” OR “cerebral gliomas”) (Topic) and (“machine learning” OR “deep learning” OR “artificial intelligence” OR “radiomics”) (Topic). The document types mainly include articles, review, editorial material, proceeding paper, meeting abstract, and letters.

### Statistical analysis

All records and cited references from web of science were analyzed by bibliometrix package of Rstudio software (version 1.4.1717). The “annual scientific production” function was used to plot the figure of the annual production of the publications. The “social structure function” was used to plot collaboration network and collaboration world map of different countries. The “country scientific production” function was used to plot the figure showing the amount of the publications of different countries. The “most cited countries” was used to plot the figure showing the rank of countries whose publications have been cited. The “three fields plot” function was drawn to show the relationship among between the countries, journals, and keywords of the publications. The “trend topics” function was used to plot the figure showing the top keywords plus (from web of science) in the past years. The “most relevant sources” function was used to plot the rank of the number of publications of the journals. The “most global cited documents” function was used to plot the rank of times that the documents were cited. The “Biblioshiny” function was applied to perform the above functions.

## Results

### Annual publication

**Figure 1:**
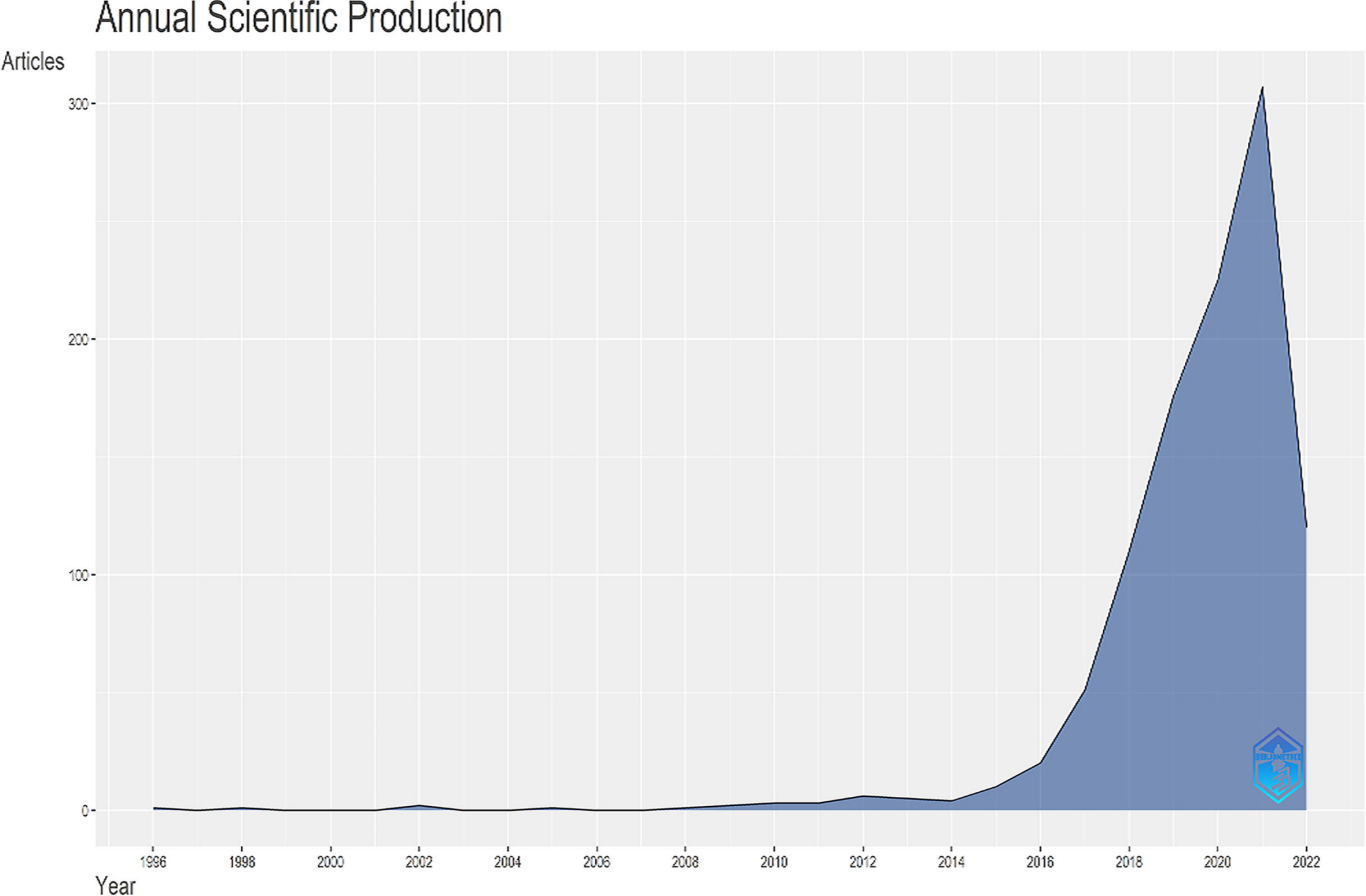
The annual scientific production in the research topic.

A total of 1081 publications from 1996 to 2022 were included, comprising 725 articles (67.07%), 141 proceeding papers (13.04%), 124 reviews (11.47%), 75 meeting abstracts (6.94%), 13 editorial materials (1.20%), and 3 letters (0.28%). The annual publication volume was no more than 100 until 2018. Then a significant increase was seen in the publication amount after 2016, reaching a total of more than 300 publications in 2021 (**Figure 1**). The annual growth rate was 30.47%.

### Country production

**Figure 2:**
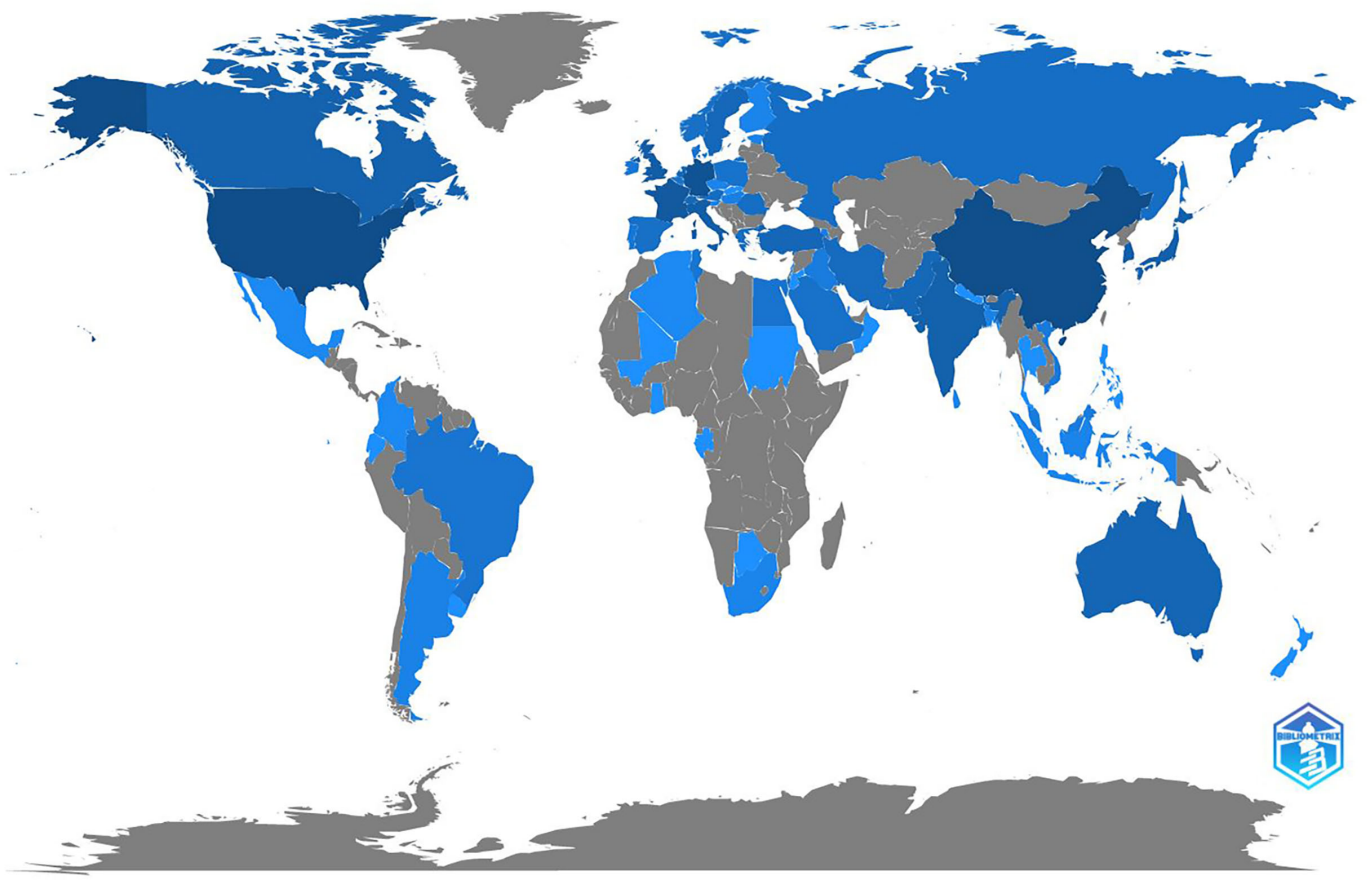
The country scientific production. The deeper the blue is, the more productive the country is.

A total of 69 countries contributed to the researches of application of AI in gliomas. The top ten productive countries were listed in **Table 1** which were the USA (1225 publications), China (1144 publications), Germany (600 publications), France (323 publications), UK (201 publications), South Korea (184 publications), Italy (172 publications), Japan (155 publications), India (151 publications), and Canada (123 publications). We can conclude from the result that Asia, Europe, and North America are the most active regions in this research field. Country scientific production was plotted in **Figure 2**. The darker the blue is, the more productive the country is.

**Table 1:** Country production rank.

Rank	Country	Production
1	USA	1225
2	China	1144
3	Germany	600
4	France	323
5	UK	201
6	South Korea	184
7	Italy	172
8	Japan	155
9	India	151
10	Canada	123

### Mapping scientific collaboration

**Figure 3:**
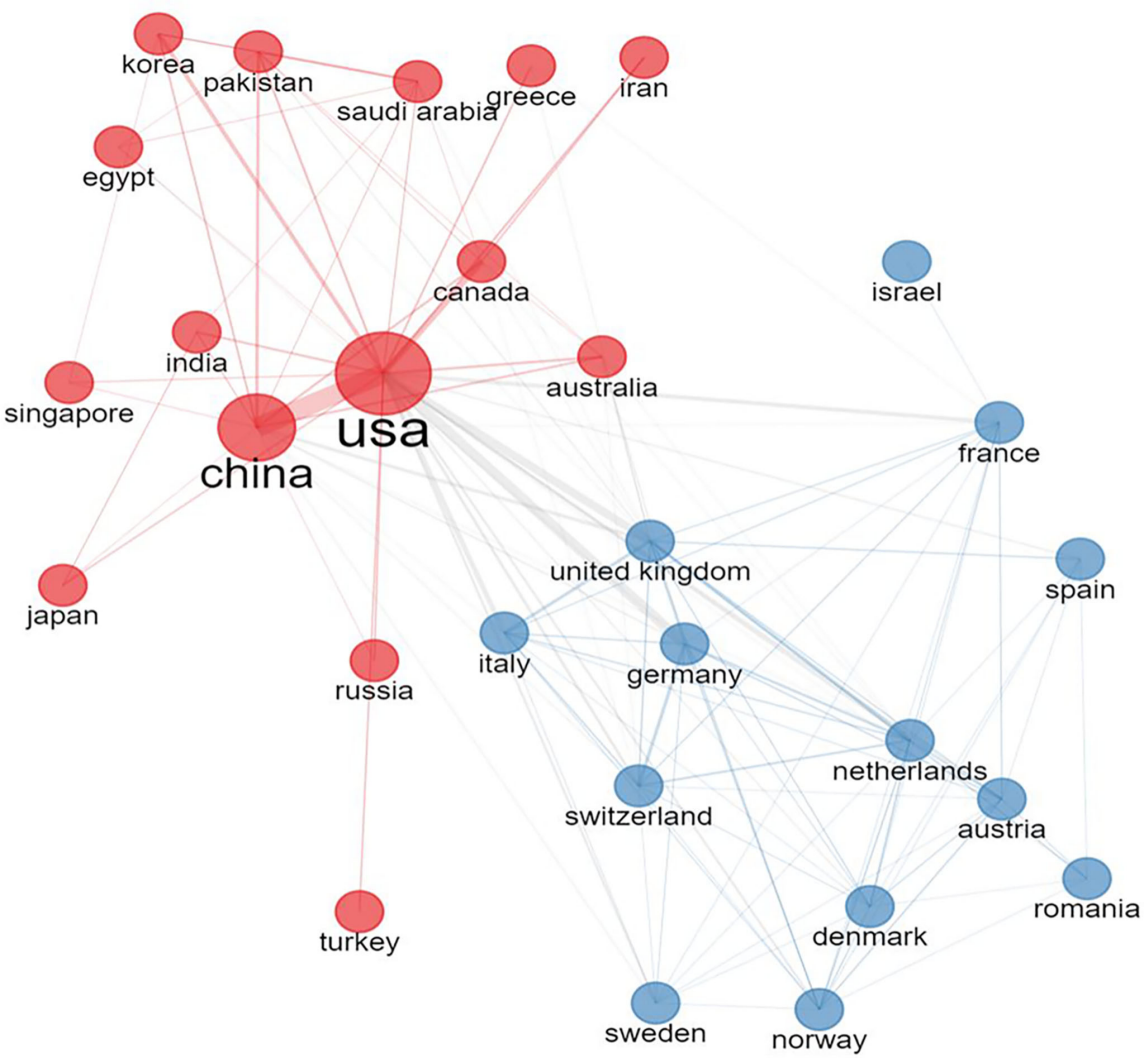
The collaboration network of different countries. All countries were divided into 2 groups according to collaborative strength. The larger the node is, the more productive the country is. The wider the line is, the stronger the collaboration is.

**Figure 4:**
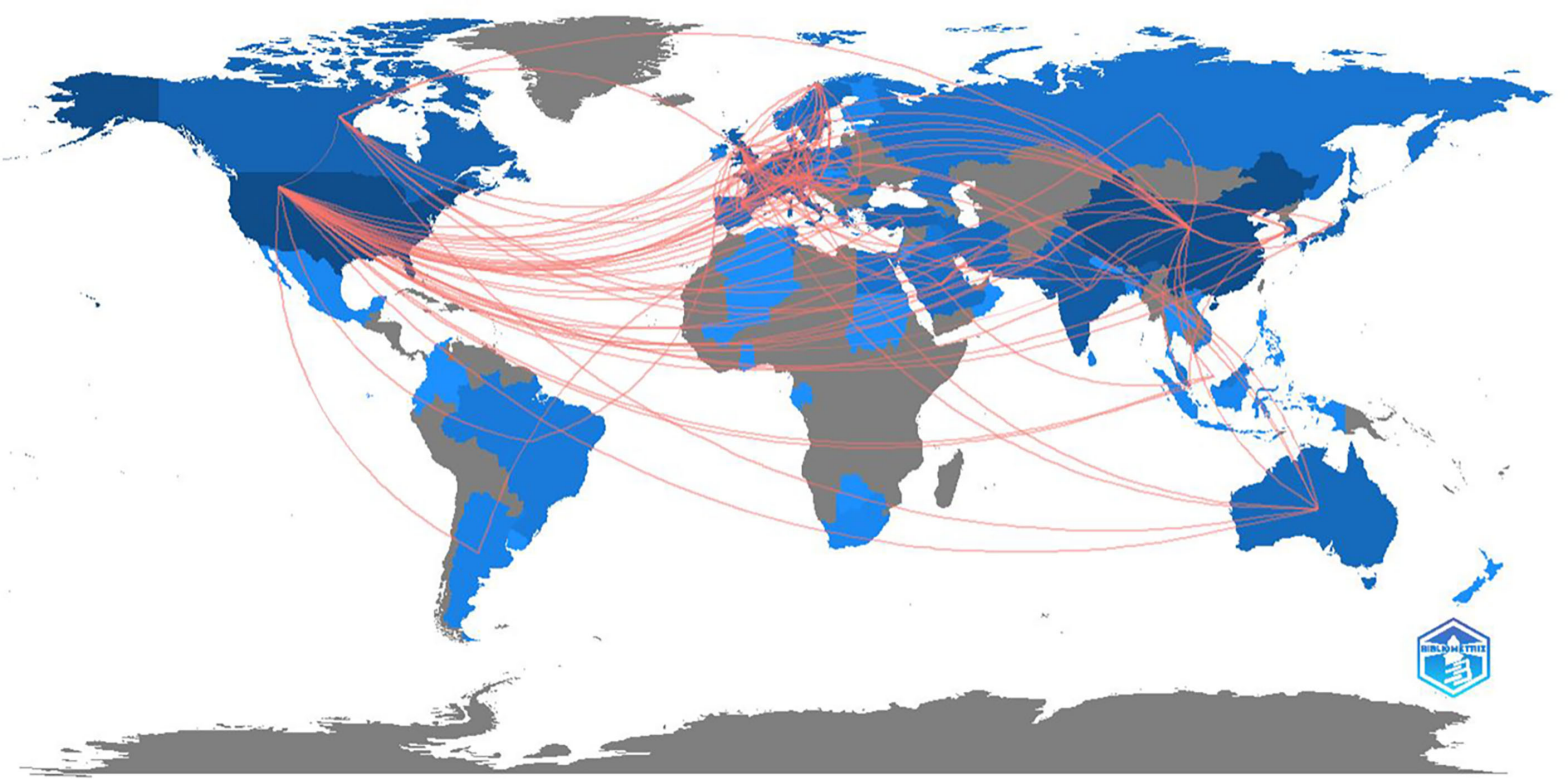
The collaboration network on world map.

In total 28 countries were included with international collaboration in their researches. The USA and China had the strongest link of international collaboration (20.09%, 9.20%, respectively) (**Figures 3**, **4**). France and South Korea had a high number of publications but their international collaboration was poor (3.92%, 2.53%, respectively).

### Trend topics and keywords

**Figure 5:**
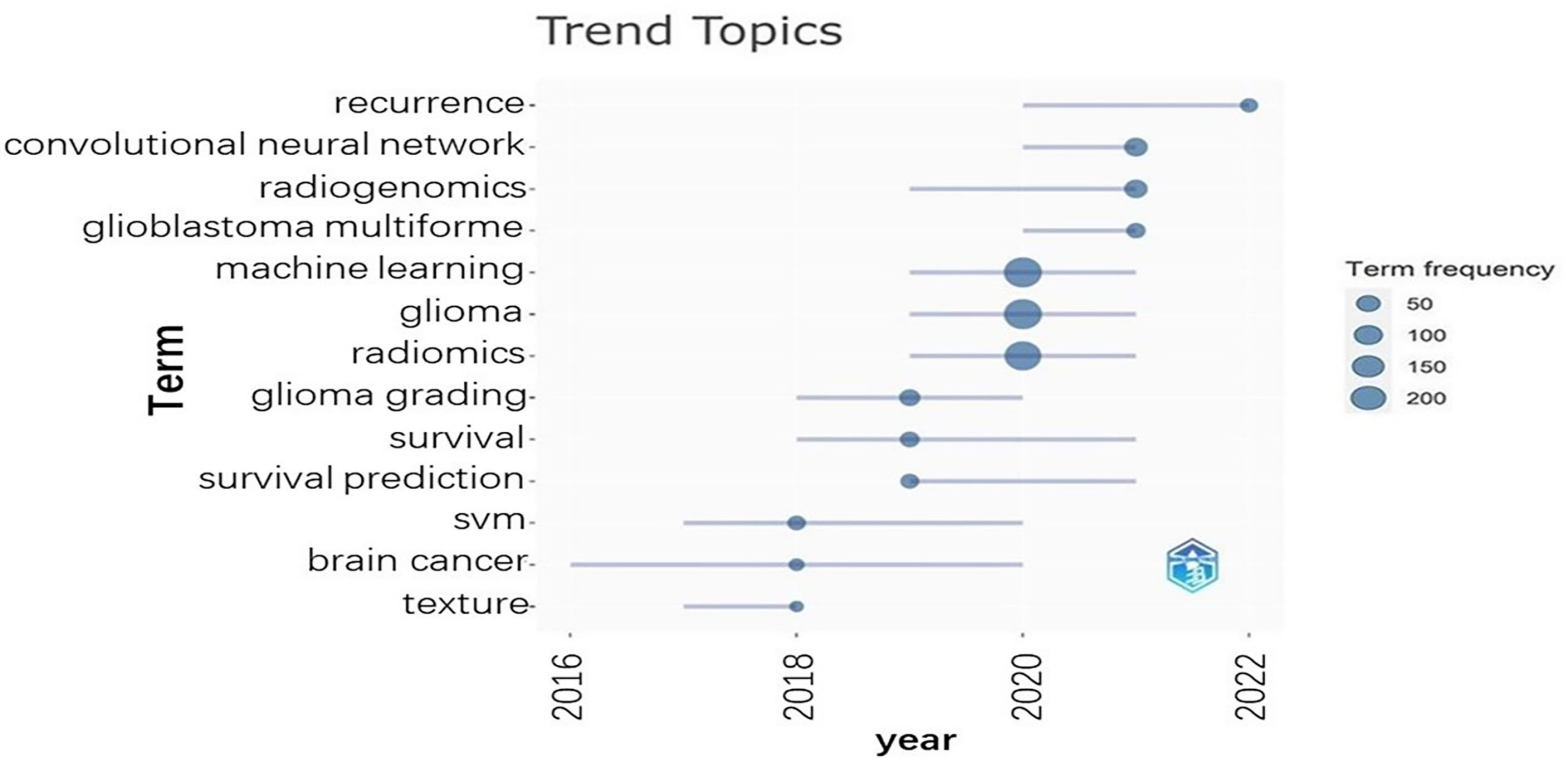
The horizontal axis is the year. The vertical axis represents the topics. The dots represent the term frequencies.

**Figure 6:**
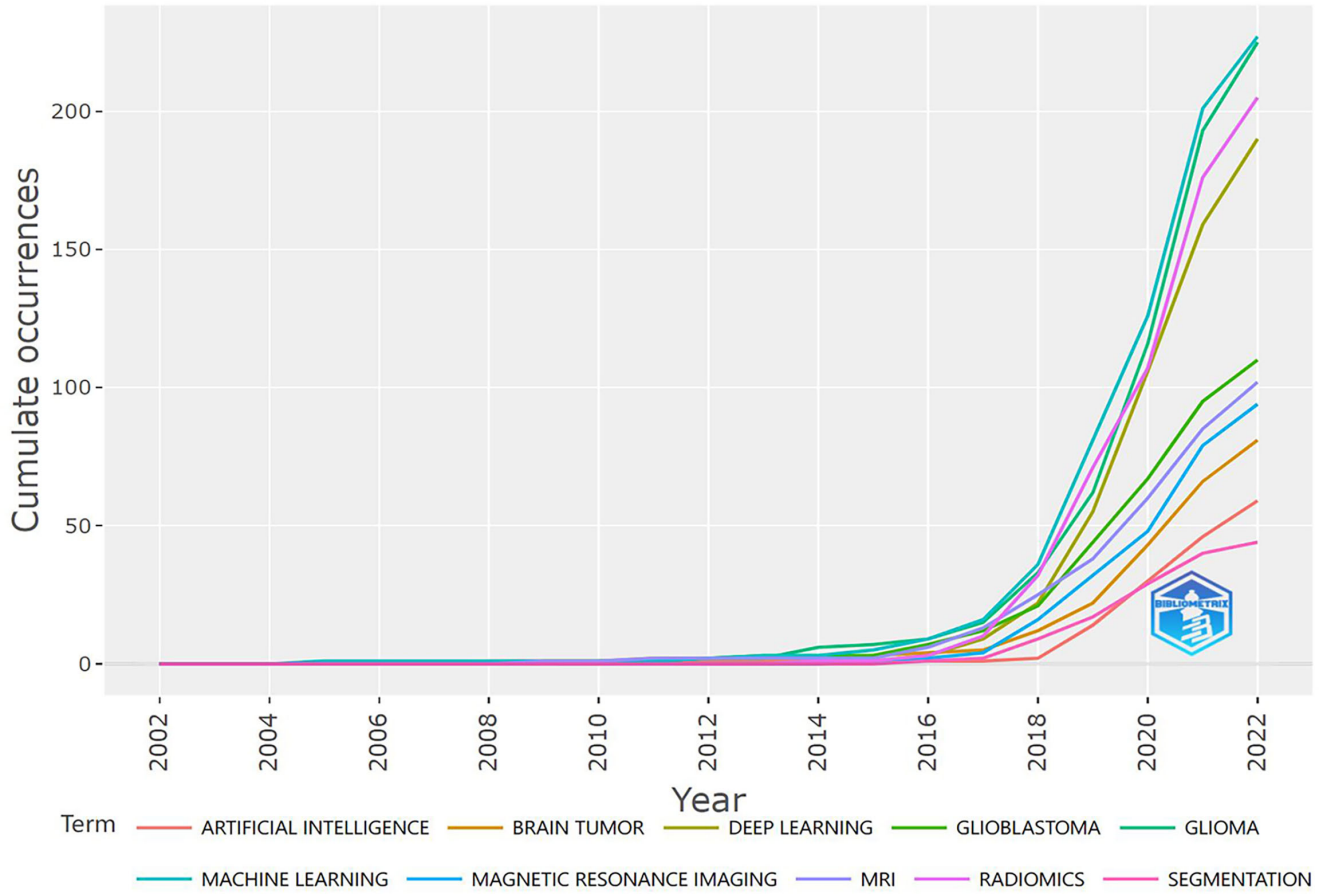
The horizontal axis represents the year. The vertical axis represents the cumulative occurrence of different terms. Different colors represent different terms.

We can conclude from **Figure 5** that several keywords have been emerging in recent years, such as convolutional neural network, radiogenomics, machine learning, and radiomics. **Figure 6** shows the cumulative occurrences of keywords in the recent years. We can conclude from **Figure 6** that the occurrences of machine learning, deep learning, and radiomics has increased significantly since 2018.

### Relevant sources

**Figure 7:**
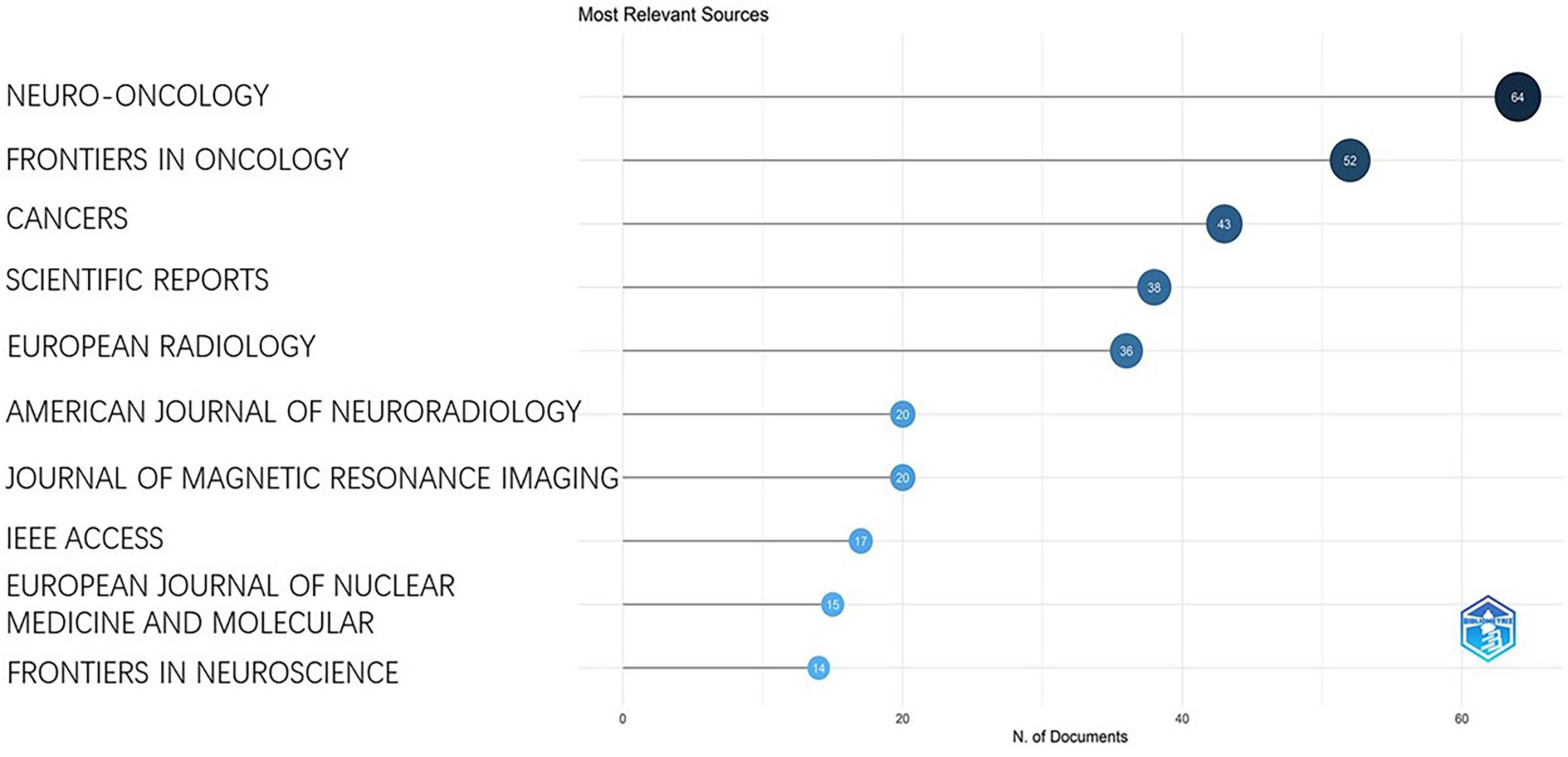
It shows the number of publications in different journals.

In the past 26 years, a total of 398 journals published articles on this research topic. The top 10 most relevant sources each has published at least 14 articles (**Figure 7**). The most represented journals were “Neuro-oncology”, “Frontiers in Oncology”, and “Cancers” with a number of publications of 64, 52, and 43, respectively.

**Figure 8:**
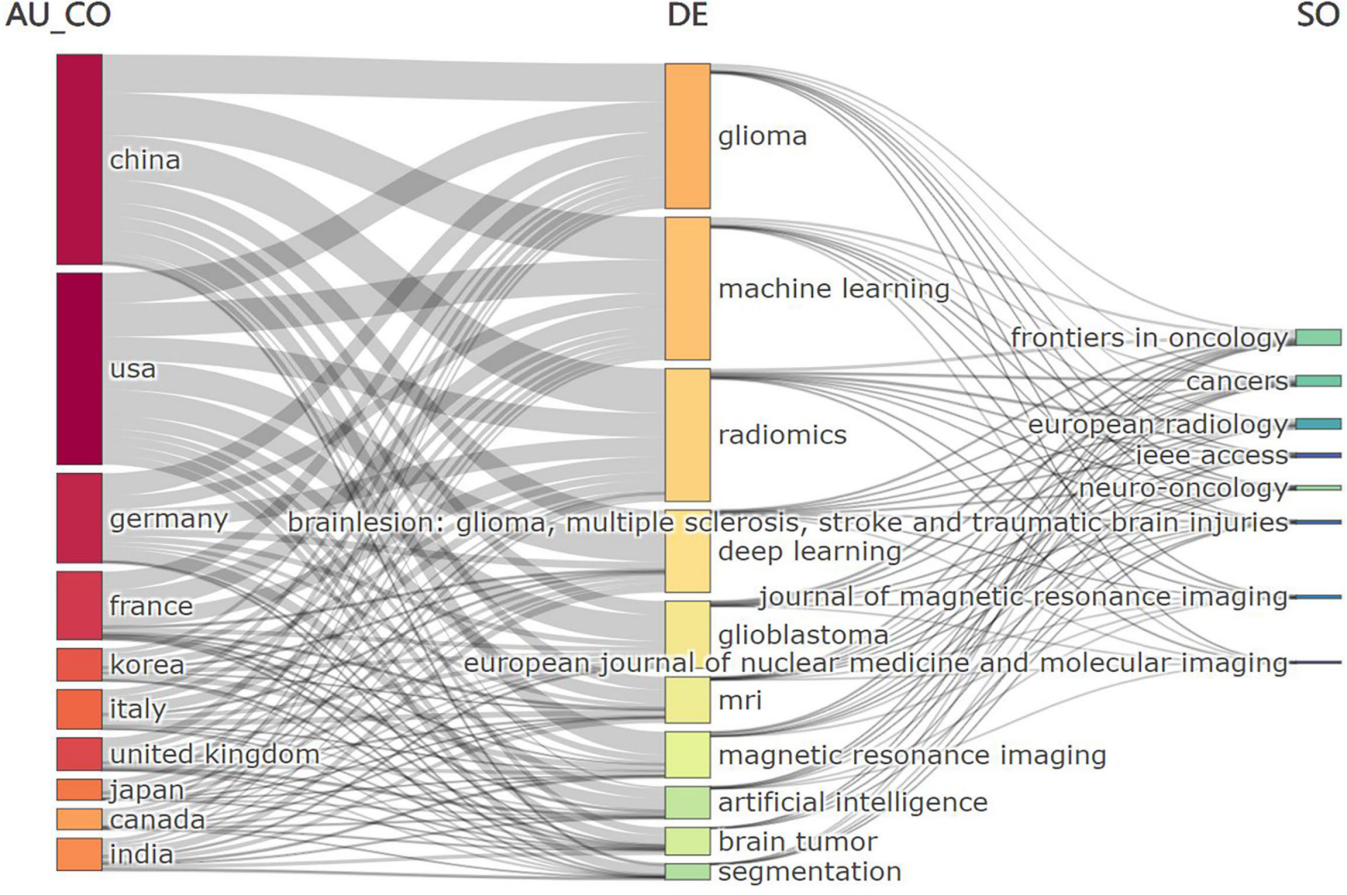
The left column represents countries. The middle column represents keywords of the publications. The right column represents journals.

The three-field plot in **Figure 8** shows the relationships between the most prolific countries, the relevant keywords, and sources. The analysis of the top 10 countries indicated that researchers from the 10 countries had strong relationships with the main topics (machine learning, deep learning, and radiomics) and they have frequently published articles in the high-ranked journals.

### Affiliations and citations

**Figure 9:**
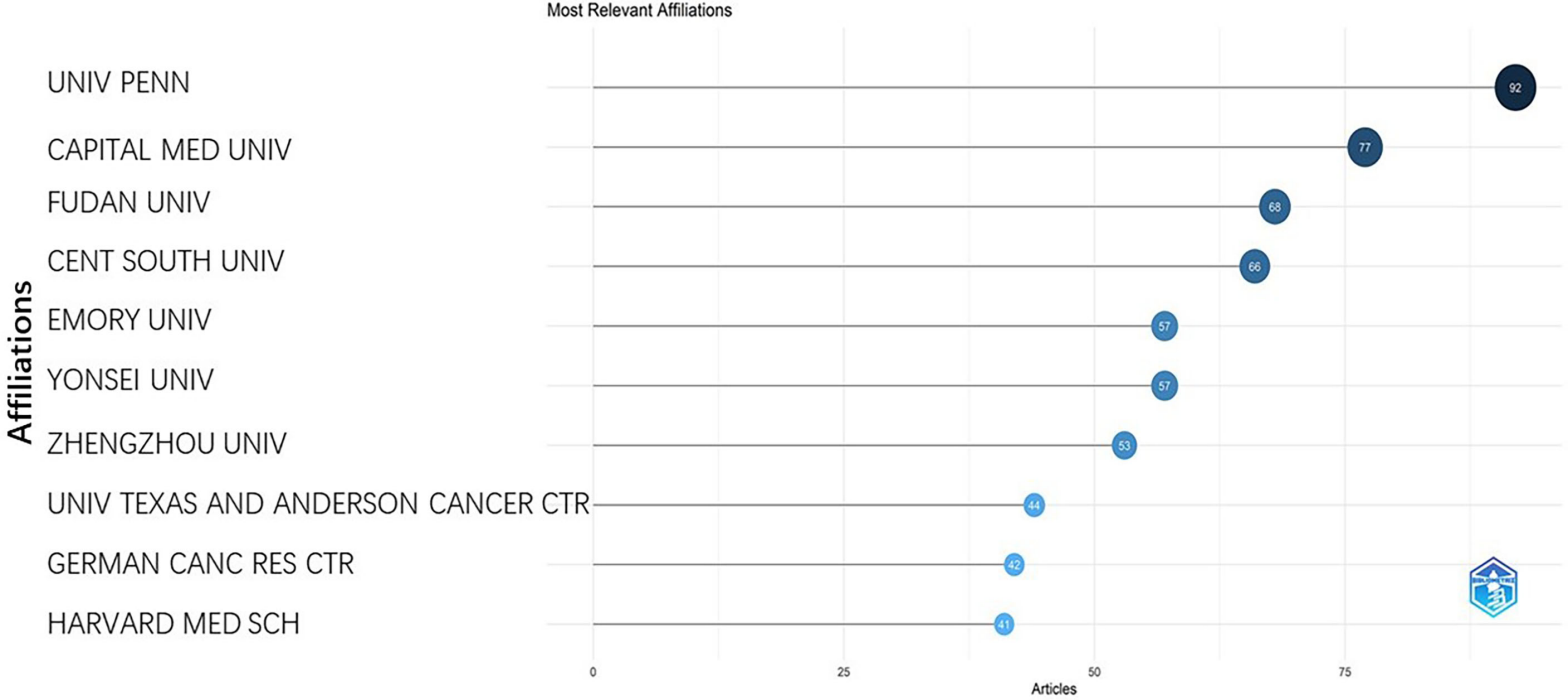
It represents the number of publications of different institutions.

**Figure 10:**
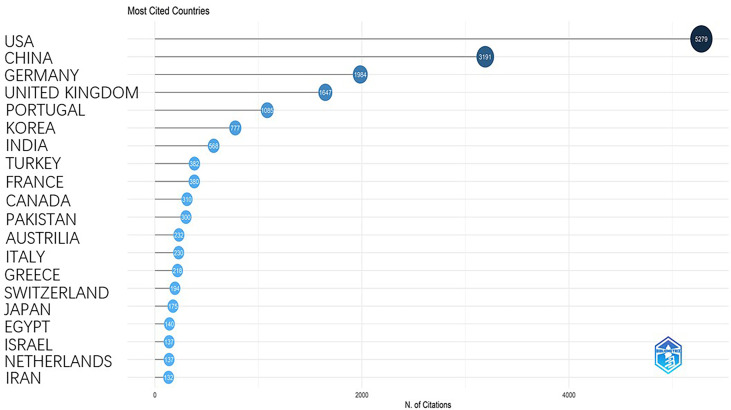
It represents the rank of cited publications of different countries.

The most prolific institution was the university of Pennsylvania, USA with a total of 92 publications, followed by Capital Medical University, China (77) and Fudan University, China (68) (**Figure 9**). The top 10 most-cited papers were listed in **Table 2** (15–24). Two publications are related to tumor segmentation. Four publications were related to the classification of tumor subtypes. Three are review articles concerning the application of AI in brain tumor. One publication is related to disease outcome. The most cited paper was published by Kamnitsas et al. on 2017. This study proposed a dual pathway, three-dimensional convolutional neural network for automatic brain lesion segmentation, including gliomas. Their method is computationally efficient, which allows its adoption in a variety of research and clinical settings. They also made the source code of the implementation publicly available. The second most cited paper was published by Sérgio Pereira et al. on 2016. This study specifically focused on the automatic segmentation of gliomas which is time-consuming in precise quantitative measurements in the clinical practice. It developed a novel convolutional neural network with small kernels. The third most cited paper was published by Capper et al. on 2018. They presented a comprehensive approach for the DNA methylation-based classification of central nervous system tumors across all entities and age groups, and demonstrated its application in a routine diagnostic setting. This method used machine learning algorithms to confirm the pathology types without substantial inter-observer variability. The fourth most cited paper was published by Zacharaki on 2009. It investigated the use of pattern classification methods for distinguishing different types of brain tumors, such as primary gliomas, and also for grading of gliomas. This study dates back to more than 10 years and at that time the application of artificial intelligence was not yet a hotpot, but it does initiate the use of machine learning in glioma researches. The fifth most cited paper is a review article by Wenya on 2019. It systematically reviewed the application of artificial intelligence in glioma, mainly in the following aspects: diagnosis, tumor detection and delineation, treatment response monitoring. The most cited countries were plotted in **Figure 10**.

**Table 2:** Most cited documents.

References	Title	Total citations	Total citation per year	Article type
KAMNITSAS K, 2017, MED IMAGE ANAL	Efficient multi-scale 3D CNN with fully connected CRF for accurate brain lesion segmentation	1413	235.5	original article
PEREIRA S, 2016, IEEE T MED IMAGING	Brain Tumor Segmentation Using Convolutional Neural Networks in MRI Images	1045	149.286	original article
CAPPER D, 2018, NATURE	DNA methylation-based classification of central nervous system tumors	957	191.4	original article
ZACHARAKI EI, 2009, MAGN RESON MED	Classification of brain tumor type and grade using MRI texture and shape in a machine learning scheme	411	29.357	original article
BI WL, 2019, CA-CANCER J CLIN	Artificial intelligence in cancer imaging: Clinical challenges and applications	382	95.5	review article
MOBADERSANY P, 2018, P NATL ACAD SCI USA	Predicting cancer outcomes from histology and genomics using convolutional networks	289	57.8	original article
LIU ZY, 2019, THERANOSTICS	The Applications of Radiomics in Precision Diagnosis and Treatment of Oncology: Opportunities and Challenges	208	52	review article
EBERLIN LS, 2012, CANCER RES	Classifying human brain tumors by lipid imaging with mass spectrometry	204	18.545	original article
TEPLYUK NM, 2012, NEURO-ONCOLOGY	MicroRNAs in cerebrospinal fluid identify glioblastoma and metastatic brain cancers and reflect disease activity	193	17.545	original article
ISIN A, 2016, PROCEDIA COMPUT SCI	Review of MRI-based Brain Tumor Image Segmentation Using Deep Learning Methods	192	27.429	review article

## Discussion

There have been an increasing number of systematic and narrative reviews focusing on the application of AI in glioma researches (25–28). They highlighted the application of machine learning and deep learning technologies in tumor segmentation and prognosis prediction. Bibliometric analysis differs from traditional narrative reviews providing readers with advances in the research topic in that it mainly focuses on trend topics and research cooperation. In medical research area, bibliometric analysis has been performed with VOSviewer (29), HistCite (30), and MTI (31). Here, we performed a novel bibliometric analysis to explore the characteristics of the publications in the past 26 years and provide a panorama of the research trends.

The production and growth rates of the scientific publications mirror the development of a certain research field. The result of our analysis showed that the publication outputs increased steadily between 2010 and 2016. However, the publication outputs increased sharply since 2016. The overall increasing tendency of publication numbers indicated that researchers made an effort to apply AI in clinical practice. The potential use of AI in assisting clinical decision making and its great computing power made it favorable to be published in high-profile journals. The year of 2016 is the milestone year after which there might be so huge a breakthrough in AI that it promotes its application in medical researches.

We can conclude from **Figure 5** that researches with omics methods and deep learning methods may be the hotspots in the future. **Figure 5** also indicates that survival prediction and glioma grading have been the common interests of researchers. This may be due to the fact that survival prediction and glioma grading were important to clinical decision making and postoperative follow-up. AI -based algorithms can extract features from medical data to construct models to facilitate survival prediction, subtype classification, or glioma grading (8, 10).

The USA and China are the main forces in researches concerning the application of AI in gliomas. The rich scientific outputs of the two countries were based on the abundant scientific funding (32–35). Although the publication number of China is the equivalent to that of the USA (1225 for the USA, 1144 for China), there is a huge gap between citation numbers of the two countries (5279 for the USA, 3191 for China), suggesting that the USA has a much better academic reputation in this research field than China does. We can see from **Figure 9** that 8 out of 10 most relevant affiliations came from the USA and China which also indicates the dominant roles of the two countries in this research area.

The geographical distribution mainly covers all continents and the predominant hubs for the clinical and translational researches were the USA, China. China is the only developing country that maintain a high scientific output which may be due to the unbalanced economic development and the metropolis such as Beijing and Shanghai undertook most of the scientific tasks. We can also conclude from **Figure 3** that the USA mainly collaborate with China and other Asian countries, while European countries tend to collaborate more with European Union members. The USA and China have both high scientific production and efficient collaboration, while other countries such as Italy, France, and Germany sustain high scientific with relatively poor international collaboration.

**Figure 7** indicated that the top 10 journals out of 398 journals were the main sources of publications in the research field. Our results also indicate that journals with relatively higher impact factor tend to publish more articles, such as Neuro-Oncology, Frontiers in Oncology, and Cancers. Among the top 10 productive journals, 5 of them were recommended for tracking the cutting edge of this research field including Neuro-Oncology, Frontiers in Oncology, American Journal of Neuroradiology, Frontiers in Neuroscience, and European Radiology. Notably, Neuro-Oncology is ranked the first in both most productive and most cited journal which reflect its prestige on the research field.

Artificial intelligence has already been widely accepted in clinical researches of gliomas, while in the past decade, it is mainly used in tumor imaging analysis, tumor segmentation, diagnosis, and prognosis prediction. In the future, however, artificial intelligence should be applied in some emerging research fields. A molecular taxonomy is being defined for the most common central nervous tumors with the wide availability and decreasing cost of next-generation sequencing. It has been observed that molecular signatures confer prognostic implications beyond standard histopathologic classifications in gliomas (19). These molecular imprints increasingly guide the frequency of surveillance imaging for a tumor, patient consultation for clinical outcome and recurrence risk, and decisions on the type of treatment to administer. However, such information was largely determined only from tissue sampling of the tumor after an operation previously. In the future, artificial intelligence should be applied in these research areas.

## Limitations

There are three main limitations of the study. First, we only retrieve data from web of science. Data from Pubmed, Embase, and Scopus should also be obtained. But data from multiple sources require more sophisticated analytical methods. Our search terms may not be perfect enough to retrieve all publications in the research field. Third, researchers from China may publish their work in Chinese journals, so the contribution of Chinese researchers may have been underestimated.

## Conclusions

To the best of our knowledge, this is the first study to conduct a bibliometric analysis to assess the research trends of the application of AI. The publication outputs have increased suddenly since 2014. Neuro-Oncology was the most high-profile journal because it was ranked first in both the most productive and the most cited journals. The USA and China are the leading contributors in the research field.

## Data availability statement

The raw data supporting the conclusions of this article will be made available by the authors, without undue reservation.

## Author contributions

DZ and WZ contributed equally to the study. XG takes final responsibility of the article. All authors contributed to the article and approved the submitted version.

## Conflict of interest

The authors declare that the research was conducted in the absence of any commercial or financial relationships that could be construed as a potential conflict of interest.

## Publisher’s note

All claims expressed in this article are solely those of the authors and do not necessarily represent those of their affiliated organizations, or those of the publisher, the editors and the reviewers. Any product that may be evaluated in this article, or claim that may be made by its manufacturer, is not guaranteed or endorsed by the publisher.

## Abbreviations

ML, machine learning; CNS, central nervous tissue; WHO, world health organization; IDH, isocitrate dehydrogenase; AI, artificial intelligence; DL, deep learning
